# Spectrophotometric determination of etodolac in pure form and pharmaceutical formulations

**DOI:** 10.1186/1752-153X-2-7

**Published:** 2008-04-14

**Authors:** Ayman A Gouda, Wafaa S Hassan

**Affiliations:** 1 Chemistry Department, Faculty of Science, Zagazig University, Zagazig, Egypt; 2 Department of analytical Chemistry, Faculty of Pharmacy, Zagazig University, Zagazig, Egypt

## Abstract

**Background:**

Etodolac (ETD) is a non-steroidal anti-inflamatory antirheumatic drug. A survey of the literature reveals that there is no method available for the determination of ETD in pure form and pharmaceutical formulations by oxidation-reduction reactions.

**Results:**

We describe three simple, sensitive and reproducible spectrophotometric assays (A-C) for the determination of etodolac in pure form and in pharmaceutical formulations. Methods A and B are based on the oxidation of etodolac by Fe^3+ ^in the presence of *o*-phenanthroline (*o*-phen) or bipyridyl (bipy). The formation of the tris-complex on reaction with Fe^3+^-*o*-phen and/or Fe^3+^-bipy mixtures in acetate buffer solution at optimum pH was demonstrated at 510 and 520 nm with *o*-phen and bipy. Method C is based on the oxidation of etodolac by Fe^3+ ^in acidic medium, and the subsequent interaction of iron(II) with ferricyanide to form Prussian blue, with the product exhibiting an absorption maximum at 726 nm. The concentration ranges are 0.5–8, 1.0–10 and 2–18 μg mL^-1 ^respectively for methods A, B and C. For more accurate analysis, Ringbom optimum concentration ranges were calculated, in addition to molar absorptivity, Sandell sensitivity, detection and quantification limits.

**Conclusion:**

Our methods were successfully applied to the determination of etodolac in bulk and pharmaceutical formulations without any interference from common excipients. The relative standard deviations were ≤ 0.76 %, with recoveries of 99.87 % – 100.21 %.

## Background

**Scheme 1:**
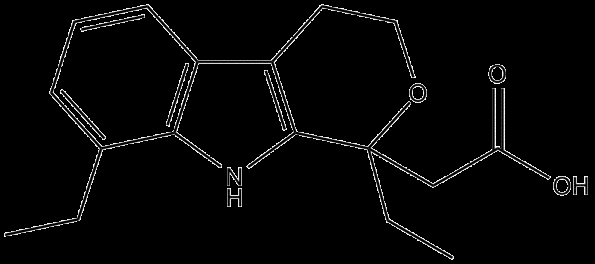
The chemical structure of Etodolac (ETD).

Etodolac (ETD), 1,8-diethyl-1,3,4,9-tetrahydropyrano- [3,4-b]indole-1-acetic acid [1], is a non-steroidal anti-inflamatory antirheumatic drug (Scheme 1). A survey of the literature reveals that there are very few reported methods for the determination of ETD in biological fluids, pharmaceutical formulations and in presence of its enantiomer. Of those studies reported, the techniques used include chromatography, HPLC [2-5], GC [6-8], in addition to spectrofluorimetric [9] and spectrophotometric methods [9-11]. However, an extensive survey of the literature revealed that there is no method available for the simultaneous determination of ETD in pure form and pharmaceutical formulations by oxidation-reduction reactions.

The aim of this study was to apply redox reactions in developing simple, accurate, sensitive and reproducible assays to analyse ETD in pure form and pharmaceutical formulations, by employing iron(III) with *o*-phenanthroline (*o*-phen), bipyridyl (bipy) and ferricyanide. This study describes spectrophotometric methods that can be used in laboratories where modern and expensive equipment, such as that required for GC or HPLC, is not available.

## Results and discussion

### Methods A and B

#### Absorption spectra

**Figure 1:**
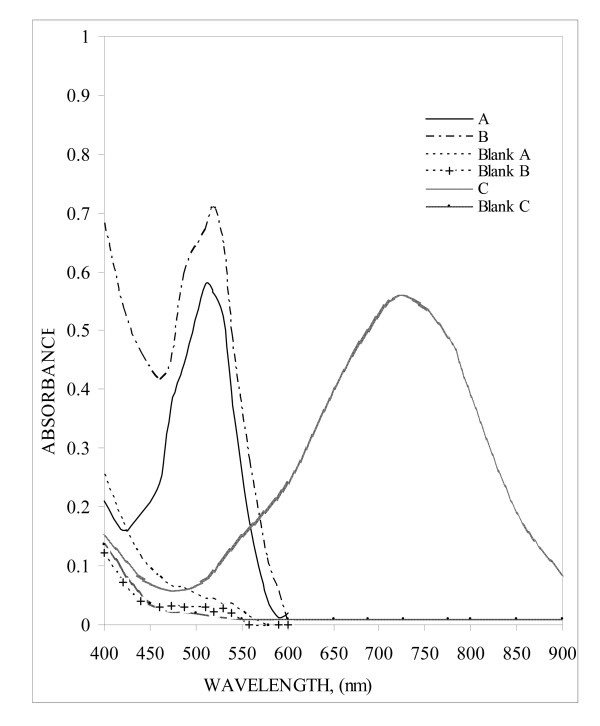
Absorption spectra of: (A) Fe(III)-1,10-phenanthroline with ETD (6.0 μg mL^-1^); (B) Fe(III)-2,2'-bipyridyl with ETD (6.0 μg mL^-1^) and (C) Fe(III)- ferricyanide with ETD (6.0 μg mL^-1^) *versus *reagent blanks for each method.

1, 10-Phenantholine and 2,2'-bipyridyl are organic bases whose chemical structures are similar, and contain the iron(II) specific group (18). Methods A and B are based on the formation of tris(o-phenanthroline)-iron(II) or tris(2,2'-bipyridyl)-iron(II) following the reaction of ETD with Fe^3+^-o-phen or Fe^3+^-bipy respectively. The reaction proceeds through the reduction of Fe^3+ ^to Fe^2+^, with the subsequent formation of an intense orange-red colouration attributable to the complex (12–20). The absorption spectra of the coloured complexes under optimum conditions were scanned in double beam mode against a reagent blank over the range 400–900 nm, and recorded according to general procedures. Characteristic λ_max _values were obtained at 510 and 521 nm for methods A and B, respectively (Figure 1). The experimental conditions were established by varying each parameter individually (21) and observing the effect on the absorbance of the coloured species. All the spectral characteristics, as well as measured or calculated factors and parameters, are summarised in Table 1.

**Table 1:** Quantitative parameters for methods A-C.

**Parameter**	**A**	**B**	**C**
λ_max_, nm	510	521	726
Beer's conc. Range (μg mL^-1^)	0.5–8.0	1.0–10	2.0–18
Ringbom conc. Range (μg mL^-1^)	0.85–7.5	2.0–8.5	3.0–16.5
Detection limits (μg mL^-1^)	0.065	0.104	0.228
Quantification limit (μg mL^-1^)	0.217	0.347	0.76
Molar absorpitivity × 10 ^4^(L mol^-1 ^cm^-1^)	1.812	1.876	1.039
Sandell sensitivity (ng cm^-2^)	15.86	15.32	27.66
Regression equation ^a^			
*S*_*y*/*x*_	0.202	0.285	0.246
Intercept	0.0078	- 0.0018	0.0011
*S*_*a*_	0.142	0.201	0.2007
± t*S*_a_	0.365	0.517	0.516
Slope	0.0582	0.066	0.036
*S*_*b*_	0.0285	0.0365	0.018
± t*S*_b_	0.0733	0.094	0.0462
Correlation coefficient (*r*)	0.9999	0.9999	0.9997
Mean ± SD%	99.87 ± 0.659	100.21 ± 0.727	99.875 ± 0.759
RSD%	0.66	0.73	0.76
Variance	0.434	0.528	0.576
SE	0.269	0.297	0.31
Student, s *t*-value^b ^(2.571)	1.16	0.323	1.07
Variance ratio *F*-test ^b ^(6.256)	1.06	1.29	1.406

^a ^A = a + b c, where c is the concentration in μg mL^-1^.

^b ^The theoretical t- and F-values, 2.571 and 6.256, respectively for five degree of freedom at 95% confidence level.

#### Effect of pH

Of the buffers investigated, acetate solution proved to be the optimal (universal, phosphate, thiel, borate and acetate). A pH adjustment was necessary especially given the acidic medium, because the reaction was affected by change in pH over the range 2.5–5.6. The optimum pH was 4.5 for both methods A and B; moreover, 4.0 mL of buffer solution was sufficient for complete colour development.

#### Effect of reagent concentration

The addition of 1.0 mL of Fe^3+^-*o*-phen (method A) or 1.5 mL of Fe^3+^-bipy (method B) solutions was sufficient to obtain the maximum and reproducible absorbance for 6.0 μg mL^-1 ^of ETD. Smaller amounts resulted in incomplete complex formation. Increased concentration had no effect on complex formation, although absorbance increased slightly owing to the reagent background used.

#### Effects of temperature and heating time

The effects of temperature and heating time on the formation of the coloured complex were also studied. The reaction of ETD with both reagents proceeded very slowly at room temperature, with higher temperature employed to accelerate the reaction. Maximum absorbance was obtained following heating on a water bath at 80°C for about 10 min with both Fe^2+^-phen and Fe^2+^-bipyridyl coloured complexes. Further heating caused no appreciable change in the colour. The complex obtained was highly stable for more than 12 h.

### Method C

#### Absorption spectra

The formation of the Prussian blue (PB) complex was employed in the qualitative detection of Fe (II). A deep blue complex was formed from the reaction between Fe(II) and hexacyanoferrate (III). As shown in Figure 1, the chromogenic reagent blank (Fe(III) mixed with hexacyanoferrate (III) in acidic medium) did not show strong absorption in the visible region of the spectrum. However, after addition of the acidic hydrolysis product of ETD, the spectrum changed because of the formation of the PB complex, which has a λ_max _of 726 nm.

The first step is the oxidation of Fe(II):

Fe^2+ ^+ [Fe(CN)_6_]^3- ^→ Fe^3+ ^+ [Fe(CN)_6_]^4-^

The second step is the formation of hexacyanoferrate (II) complex (PB):

4Fe^3+ ^+ 3 [Fe(CN)_6_]^4-^+ acidic hydrolysis product of (ETD) → Fe_4 _[Fe(CN)_6_]_3_

The complex formed is highly insoluble (K*sp *= 3 × 10^-41^) (22). Neither iron(III) nor ferricyanide solution absorb at 726 nm. Hence, the use of measured volumes of reagent, and measurement against the corresponding reagent blank gave a linear calibration for the drug. We herefore report the formation and application of the Prussian blue complex in the development of a sensitive spectrophotometric method for the determination of ETD.

The optimum conditions were established by varying parameters, such as iron(III), ferricyanide and acid concentrations, reaction time and the order in which the reagents were added:

#### Optimum iron(III) and ferricyanide concentrations

Having studied the effect of iron(III) chloride concentration on colour development, it was observed that the absorbance increased as the volume of 0.2% iron(III) solution increased, reaching a maximum upon the addition of 2.0 mL of the 0.2% iron(III) solution to 6.0 μg mL^-1 ^of ETD and 2.0 mL of 0.2% ferricyanide solution in a total volume of 10 mL. These results indicate that maximum absorbance was obtained when the final iron(III) chloride concentration was 0.04%. Larger volumes of iron(III) chloride (up to 4.0 mL) had no effect on the sensitivity of the reaction. Similar observations were made when varying volumes of 0.2% ferricyanide solution were added to fixed amounts of ETD (6.0 μg mL^-1^) and iron(III) chloride (2 mL; 0.2%), and diluted to 10 mL after full colour development. The results of this study reveal that the concentrations of iron(III) and ferricyanide reagents are not critical. However, 2.0 mL of 0.2% reagent solutions in a total volume of 10 mL were used to ensure adequate reagent concentrations for higher drug concentrations.

#### Effect of nature of acid, and its concentration

The reaction product, Prussian blue, was found to flocculate within 20–30 min of colour development. To delay the flocculation, acid was added after full colour development and before dilution. Sulphuric acid was found to give more stable colour and reproducible results compared to hydrochloric acid. A 1.0 mL volume of 10 M sulphuric acid in a total volume of 10 mL was found to be adequate.

#### Effect of reaction time and stability of coloured species

The reaction is slow at 30 ± °2°C, but absorbance increased with time and reached a maximum in 10 min, with colouration remaining stable for at least 6 h.

#### Effect of order in which reagents were added

After fixing all other parameters, a few other experiments were performed to ascertain the influence of the order in which reagents were added. The following order: drug, ferricyanide and iron(III), followed by sulphuric acid after full colour development, gave maximum absorbance and stability, and for this reason the same order of addition was followed throughout the investigation.

### Method validation

#### Linearity

Under the optimum conditions described, Beer's law holds over the concentration ranges 0.5–8, 1–10 and 2–18 μg mL^-1 ^respectively for methods A, B and C. The optimum concentration ranges of ETD that can be measured accurately, as evaluated from the Ringbom plot, are 0.85–7.5, 2.0–9.0 and 3.0–16.5 μg mL^-1 ^using methods A, B and C, respectively. We calculated the apparent molar absorptivity, Sandell sensitivity, relative standard deviations for six replicate determinations of 6.0 μg mL^-1 ^and the regression equations (Table 1).

#### Sensitivity

The detection limit (LOD) for the proposed methods was calculated using the following equation according to IUPAC definition (23):

LOD = *3s/k*;

where *s *is the standard deviation of replicate determination values under the same conditions as the sample analysis in the absence of the analyte, and *k *is the sensitivity, namely the slope of the calibration graph. In accordance with the formula, the detection limits obtained for the absorbance were found to be 0.065, 0.104 and 0.228 μg mL^-1 ^for the ETD-Fe^2+^-phen, Fe^2+^-bipyridyl and Fe^2+^-ferricyanide methods, respectively.

The limits of quantitation, LOQ, is defined as;

LOQ = *10 s/k*

According to this equation, the LOQs were found to be 0.217, 0.347 and 0.76 μg mL^-1 ^for the Fe^2+^-phen, Fe^2+^-bipyridyl and Fe^2+^-ferricyanide methods, respectively.

#### Accuracy and precision

In order to determine the accuracy and precision of the proposed methods, solutions containing four different concentrations of ETD were prepared and analysed in six replicates. The relative standard deviation as precision and percentage relative error (Er %) as accuracy of the suggested methods were calculated at 95% confidence levels, and can be considered satisfactory. Precision was carried out by six determinations at four different concentrations in these spectrophotometric methods. The percentage relative error was calculated according the following equation:

Er % = [(found – added)/added] × 100

The inter- and intra-day precision and accuracy results are shown in (Table 2). The analytical results for accuracy and precision show that the methods proposed have good repeatability and reproducibility.

**Table 2:** The intra-day and inter-day precision and accuracy data for ETD obtained by the proposed methods (A – C).

		Intra-day			Inter-day		
		
Method	Added (μg mL^-1^)	Found ± SE ^a, b ^(μg mL^-1^)	Precision RSD %	Accuracy R.M.E %	Found^a, b ^(μg mL^-1^)	Precision RSD %	Accuracy R.M.E %
A	1	0.99 ± 0.45	1.11	-1.00	1.01 ± 0.20	1.49	1.00
	3	3.02 ± 0.49	1.20	0.67	2.97 ± 0.37	0.91	-1.00
	5	4.99 ± 0.26	0.64	-0.20	4.94 ± 0.31	0.77	-1.20
	7	6.97 ± 0.24	0.59	-0.43	6.94 ± 0.34	0.85	-0.86
							
B	2	2.02 ± 0.55	1.34	1.00	2.01 ± 0.63	1.54	0.50
	4	3.97 ± 0.31	0.76	-0.75	3.99 ± 0.46	1.13	-0.25
	6	5.93 ± 0.29	0.71	-1.17	5.96 ± 0.43	1.06	-0.67
	8	8.05 ± 0.36	0.88	0.625	7.97 ± 0.38	0.93	-0.38
							
C	4	3.98 ± 0.18	0.45	-0.50	4.01 ± 0.46	1.12	-0.50
	8	7.95 ± 0.20	0.50	-0.625	8.03 ± 0.40	0.98	0.38
	12	11.94 ± 0.33	0.82	-0.50	12.04 ± 0.48	1.16	0.33
	16	16.02 ± 0.49	1.19	0.125	15.91 ± 0.54	1.32	-0.56

^a ^Average of six determinations.

^b ^Mean ± standard error.

RSD%, percentage relative standard deviation;

R.M.E %, percentage relative mean error.

#### Effects of interference

The interference criterion was an error of not more than ± 3.0 % in the absorbance. To test the efficiency and selectivity of the proposed analytical methods (A-C) to pharmaceutical formulations, we carried out a systematic study of additives and excipients (*e.g*. lactose, glucose, dextrose, talc, calcium hydrogen phosphate, magnesium stearate and starch) that usually present in dosage forms. Experiments showed that there was no interference from additives or excipients for methods A-C (Table 3).

**Table 3:** Determination of ETD in presence of additives or excipients.

**Material**	**Amount (mg)**	**Method A**	**Method B**	**Method C**
		
		**Recovery ^a ^%± SD ^b^**	**Recovery ^a ^%± SD ^b^**	**Recovery ^a ^%± SD ^b^**
Lactose	50	99.63 ± 0.82	99.20 ± 0.78	100.2 ± 0.51
Glucose	50	98.84 ± 0.67	100.35 ± 1.22	99.55 ± 0.88
Dextrose	50	99.30 ± 0.78	98.70 ± 0.56	98.65 ± 0.46
Magnesium stearate	30	99.25 ± 0.75	99.55 ± 0.89	100.15 ± 1.30
Calcium hydrogen phosphate	50	99.50 ± 0.96	98.95 ± 0.73	99.40 ± 0.84
Talc	40	99.80 ± 0.61	100.40 ± 1.05	100.10 ± 0.95
Starch	50	100.05 ± 1.14	98.80 ± 0.69	99.75 ± 0.62

^a ^6.0 μg mL^-1 ^of ETD was taken.

^b ^Average of five determinations.

SD: Standard deviation.

The main degradation products of etodolac are identified as 7-ethyl-2(1-methylene propyl)-1H-indole-3-ethanol, 1,8 diethyl-1-methyl-1,3,4,9-tetrahydropyrano- [3,4-b]indole and 7-ethyl tryptophol [24]. Consequently, there was no interference of the degradation products in the determination of the ETD in proposed methods.

#### Analytical applications

Because our three methods were successfully applied to the determination of ETD in its pharmaceutical formulations, they could therefore be used easily for the routine analysis of pure ETD and its dosage forms. Moreover, to check the methods' validity, dosage forms [Napilac capsules (200 mg ETD per capsule) and Etodine capsules (300 mg ETD per capsule)] were tested for possible interference with the standard addition method (Table 4). The methods' performance was assessed using the t-test (for accuracy) and a variance ratio F-value (for precision) compared with the reference method [9] (for 95% confidence level with five degrees of freedom (25)). The results showed that the t- and F-values were less than the critical value, indicating that there was no significant difference between the proposed and reference method for ETD (Table 5). Because the proposed methods were more reproducible and had higher recoveries than the reference method, they can be recommended for adoption in routine analysis in the majority of drug quality control laboratories.

**Table 4:** Determination of ETD in it's pharmaceutical dosage form applying the standard addition technique.

**Dosage forms**	**Taken (μg mL^-1^)**	**Added (μg mL^-1^)**	**Proposed methods Recovery ^a ^% ± RSD**	
			
			**Napilac capsules**	**Etodine capsules**
A	2.0	-	99.98 ± 0.19	100.01 ± 0.16
		1.0	100.05 ± **0.20**	99.99 ± **0.18**
		3.0	99.93 ± 0.32	99.60 ± 0.26
		5.0	99.84 ± 0.47	100.35 ± 0.32
				
B	2.0	-	100.09 ± 0.21	99.95 ± 0.27
		2.0	99.74 ± 0.26	99.60 ± 0.18
		4.0	99.30 ± 0.38	100.10 ± 0.22
		6.0	99.55 ± 0.55	99.70 ± 0.56
				
C	4.0	-	100.25 ± 0.19	100.08 ± 0.23
		4.0	99.80 ± 0.34	100.40 ± 0.51
		8.0	100.15 ± 0.45	98.90 ± 0.49
		12	99.79 ± 0.62	100.05 ± 0.54

^a ^Average of six determinations.

**Table 5:** Determination of ETD in pharmaceutical preparations using the proposed methods.

Sample			Recovery^a ^± SD (%)		
			
		Official method	Proposed methods		
		
			A	B	C
Napilac capsules (200 mg ETD/Capsule)	X ± SD	99.70 ± 1.16	98.52 ± 0.79	100.65 ± 1.19	100.27 ± 0.92
	*t*^b^		1.87	0.74	0.86
	*F*^b^		2.17	1.05	1.60
Etodine capsules (300 mg ETD/Capsule)	X ± SD	100.50 ± 1.36	99.95 ± 0.81	100.30 ± 0.96	100.04 ± 1.02
	*t*^b^		0.78	0.27	0.61
	*F*^b^		2.82	2.01	1.78

^a ^Average of six determinations.

^b ^The theoretical t- and F-values, 2.571 and 6.256, respectively for five degree of freedom at 95% confidence level.

## Conclusion

The methods proposed are simpler, less time consuming and more sensitive than those previously published. All the proposed methods were more advantageous than other reported visible spectrophotometric [9-11] methods with respect to sensitivity, simplicity, reproducibility, precision, accuracy and stability of the coloured species for ≥ 12 h. The proposed methods are suitable for the determination of ETD in pure form and pharmaceutical formulations without interference from excipients such as starch and glucose, suggesting potential applications in bulk drug analysis.

## Experimental

### Apparatus

All absorption spectra were recorded using a Kontron 930 (UV-Visible) spectrophotometer (German) with a scanning speed of 200 nm/min and a band width of 2.0 nm, equipped with 10 mm matched quartz cells. A Hanna pH meter (USA) was used for checking the pH of buffer solutions.

### Materials and reagents

All chemicals and materials were of analytical grade, and all solutions were freshly prepared in bidistilled water.

#### Pure samples

Etodolac (ETD) pure grade was supplied by Pharco, Egypt. The purity was found to be 100.35 ± 0.64 % according to the Pharco method [26] in which the absorbance of 0.002% w/v etodolac solution in 0.1 N sodium hydroxide was measured at 276 nm.

#### Standard Stock solutions

Stock solutions of ETD were prepared by dissolving 100 mg of pure drug in methanol, followed by dilution to 100 mL with the same solvent to obtain 1 mg mL^-1^standard solutions. Working solutions were prepared by an appropriate dilution of the stock standard solution.

#### Market samples

Napilac capsules (200 mg ETD/Capsule) were provided by Global Napi Co (Egypt) and Etodine capsules (300 mg ETD/Capsule) were provided by Pharco (Egypt).

##### Reagents

1. Iron(III)-o-phenanthroline (27) was prepared by mixing 0.198 g of 1,10 phenanthroline monohydrate (Fluka, Swiss), 2 mL of 1 M HCl and 0.16 g of ferric ammonium sulphate dodecahydrate (Aldrich, Germany) before dilution with bidistilled water to 100 mL in a calibrated flask.

2. Iron(III)-bipyridyl (27) was prepared by dissolving 0.16 g of 2, 2'-bipyridyl (Fluka, Switzerland) in 2 mL of 1 M HCl and 0.16 g of ferric ammonium sulphate dodecahydrate, before dilution with bidistilled water to 100 mL in a calibrated flask.

3. Anhydrous FeCl_3 _(Merck) and K_3 _[Fe(CN)_6_] (BDH Lab. Chemicals, Poole, England) 0.2% (w/v) were prepared in bidistilled water. Sulphuric acid (10 M) was prepared by adding 555 mL of concentrated acid, (Sp. Gr. 1.83) to 445 mL of bidistilled water with cooling (28).

4. The acetate buffer solutions, with pH ranges from 2.56 – 5.6, were prepared by mixing appropriate quantities of 0.2 M sodium acetate with 0.2 M acetic acid to obtain the desired pH as previously recommended (29).

### Recommended analytical procedure

#### Methods A and B

100 μg mL^-1 ^aliquots of the standard solutions (A: 0.05–0.8 and B: 0.1–1.0 mL) were transferred to a series of 10 mL calibrated flasks. To these were added 1.0 mL of Fe^3+^-*o*-phen (method A) or 1.5 mL of Fe^3+^-bipy (method B) reagent solutions and 4.0 mL of acetate buffer (pH 4.5), before heating on a water bath at 80°C for 10 min. The mixture was cooled to room temperature (25 ± 1°C) and the volume made up to the mark with bidistilled water. The coloured complexes formed were measured at 510 and 521 nm against a reagent blank treated similarly according to methods A and B.

#### Method C

Into a series of 10 mL calibrated flasks, aliquots (0.2–1.8 mL) of 100 μg mL^-1 ^standard solutions were transferred using a micro burette and the total volume adjusted to 3 mL by adding bidistilled water. Then, 2 mL each of FeCl_3 _(0.2 %) and K_3_Fe(CN)_6 _(0.2 %) were added to each flask, mixed well and left to stand for 10 min. Finally, 1 mL of 10 M H_2_SO_4 _was added to each flask, diluted to the mark with bidistilled water and mixed well. The absorbance of the resulting solution was measured at 726 nm for ETD against a reagent blank prepared similarly. A calibration graph was constructed by plotting the absorbance against the drug concentration or calculated regression equation.

### Analysis of pharmaceutical formulations

Ten tablets were accurately weighed and powdered. An accurately weighed quantity equivalent to 20 mg ETD was dissolved in 20 mL of methanol and transferred to a 100 mL calibrated flask. The contents of the flask were shaken for 10 min, and then made up to the mark with methanol. The general procedure was then followed for concentration ranges already mentioned for methods A, B and C.
